# From dual to single umbilical artery: a case of umbilical artery thrombosis with hypercoiling and literature review

**DOI:** 10.3389/fmed.2025.1606697

**Published:** 2025-08-21

**Authors:** Weimin Ding, Jiajia Yan, Xinyu Lin, Liangqi Yan, Enfu Tao

**Affiliations:** ^1^Department of Obstetrics, Wenling Maternal and Child Health Care Hospital, Wenling, Zhejiang, China; ^2^Department of Ultrasound, Wenling Maternal and Child Health Care Hospital, Wenling, Zhejiang, China; ^3^Department of Neonatology and NICU, Wenling Maternal and Child Health Care Hospital, Wenling, Zhejiang, China

**Keywords:** umbilical artery thrombosis, single umbilical artery, fetal growth restriction, prenatal ultrasound, hypercoiling, abnormal cord length

## Abstract

Umbilical artery thrombosis (UAT) is an extremely rare but severe obstetric complication associated with adverse perinatal outcomes, including fetal growth restriction (FGR), fetal distress, and intrauterine fetal demise. This case report highlights the diagnostic challenges of UAT and its potential misdiagnosis as a single umbilical artery (SUA). A 32-year-old woman with a history of uncomplicated vaginal delivery was initially misdiagnosed with SUA at 29 3/7 weeks of gestation. At 32 1/7 weeks, detailed ultrasound examination revealed a single umbilical artery, FGR, and hypercoiling. Following a multidisciplinary evaluation and administration of fetal lung maturation therapy, a cesarean section was performed at 32 3/7 weeks of gestation. Intraoperatively, the umbilical cord measured 90 cm in length (normal range: 35–70 cm) with over 50 coils and exhibited localized discoloration. A male neonate weighing 1,490 g (5th percentile for gestational age) was delivered with Apgar scores of 10/10 at 1 and 5 minutes. Postnatal pathological examination confirmed UAT with arterial occlusion and hypercoiling. The neonate was treated in the neonatal intensive care unit (NICU) for respiratory distress syndrome (RDS) and prematurity-related complications, achieving a favorable outcome with discharge at 27 days and normal developmental follow-up. This case underscores the diagnostic challenges of differentiating UAT from SUA and emphasizes the importance of meticulous prenatal ultrasound evaluation, particularly in cases of FGR. The hypercoiling observed in this case is exceptionally rare and may have contributed to the thrombotic event. Early recognition, timely intervention, and multidisciplinary management are critical to optimizing maternal and neonatal outcomes. This report also provides a comprehensive literature review on the etiology, diagnostic strategies, and management of UAT, highlighting the role of ultrasound and pathological examination in accurate diagnosis. The findings suggest that hypercoiling and abnormal cord length may be significant risk factors for UAT, warranting further investigation into their pathophysiological mechanisms and clinical implications.

## Introduction

Umbilical artery thrombosis (UAT) is an extremely rare but severe obstetric complication associated with adverse perinatal outcomes, including fetal growth restriction (FGR), fetal distress, and intrauterine fetal demise (1–4). The etiology of UAT remains unclear, but studies suggest it may be related to structural abnormalities of the umbilical cord (e.g., excessive length, thinness, or hypercoiling) (5), mechanical cord injuries (e.g., true knots or vascular punctures) (6), or maternal and fetal pathological conditions (e.g., gestational diabetes, hypercoagulability, or infections) (7). Prenatal diagnosis of UAT is challenging and primarily relies on ultrasound imaging, though it must be differentiated from a single umbilical artery (SUA). Misdiagnosis of UAT as SUA has been reported to lead to perinatal mortality (4, 8). Early identification, close monitoring, and timely delivery are crucial to preventing adverse outcomes.

This report presents a case of UAT initially misdiagnosed as SUA at an external hospital during the third trimester. The correct diagnosis at our institution prompted immediate hospitalization. Due to FGR and chronic fetal distress, a cesarean section was performed after fetal lung maturation therapy. Intraoperatively, the umbilical cord measured 90 cm with over 50 coils and exhibited localized discoloration. Both maternal and neonatal outcomes were favorable, with pathology confirming thrombosis in one umbilical artery. This case highlights the diagnostic challenges of UAT and the importance of timely intervention to optimize outcomes.

## Case description

**Figure 1:**
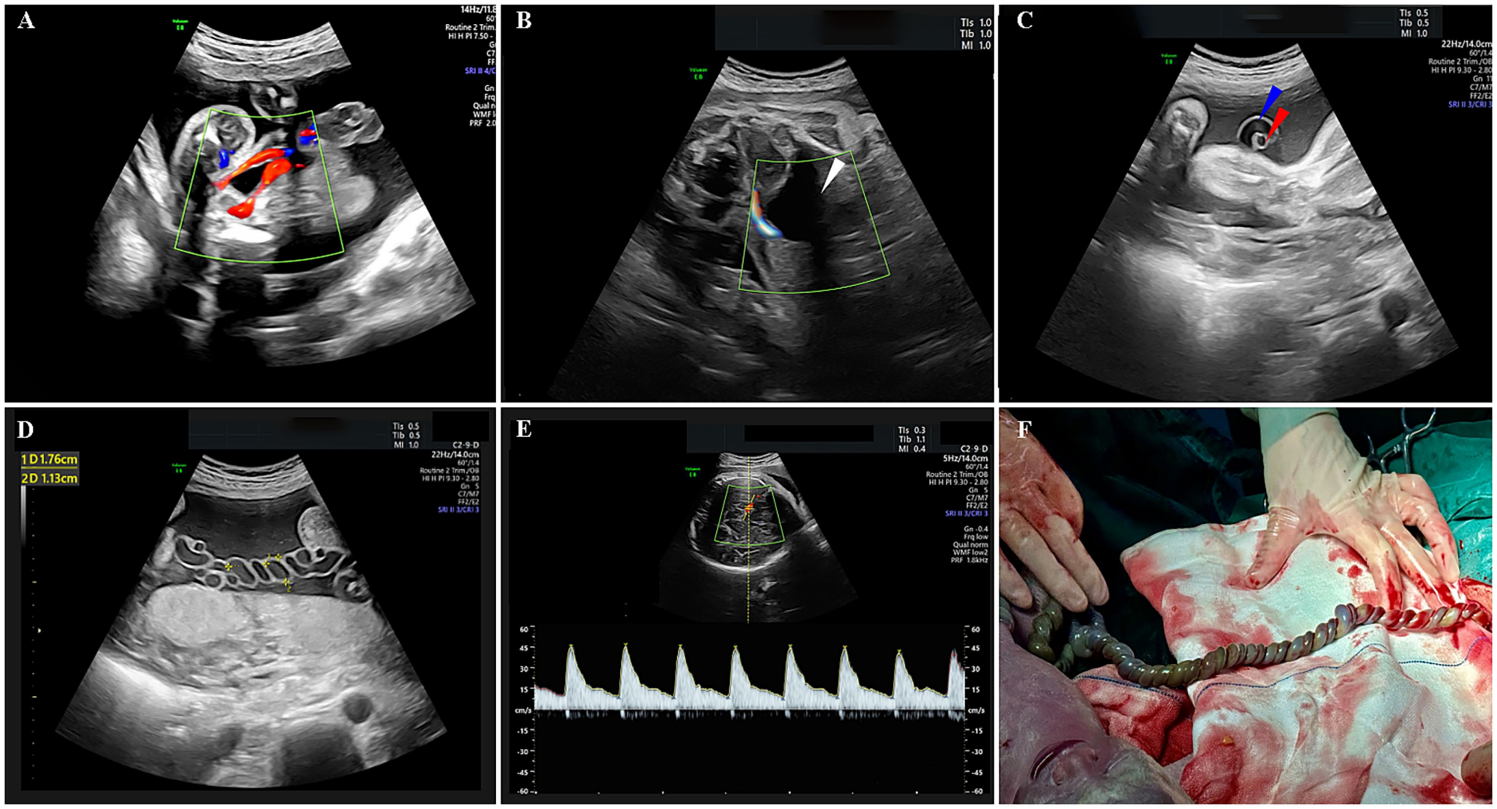
Prenatal ultrasound and post-delivery gross findings. **(A)** Prenatal ultrasound showing umbilical artery blood flow on both sides of the bladder at 23 3/7 weeks of gestation. **(B)** Prenatal ultrasound showing umbilical artery blood flow on only one side of the bladder at 32 1/7 weeks of gestation. **(C)** One umbilical artery with umbilical vein in cross-section. **(D)** Hypercoiling of the umbilical cord. **(E)** Blood flow of the middle cerebral artery. **(F)** Hypercoiled umbilical cord with focal discoloration. An umbilical cord measuring 90 cm in length with over 50 coils was observed. The white arrow indicates the bladder, the blue arrow indicates the umbilical vein, and the red arrow indicates the umbilical artery.

**Figure 2:**
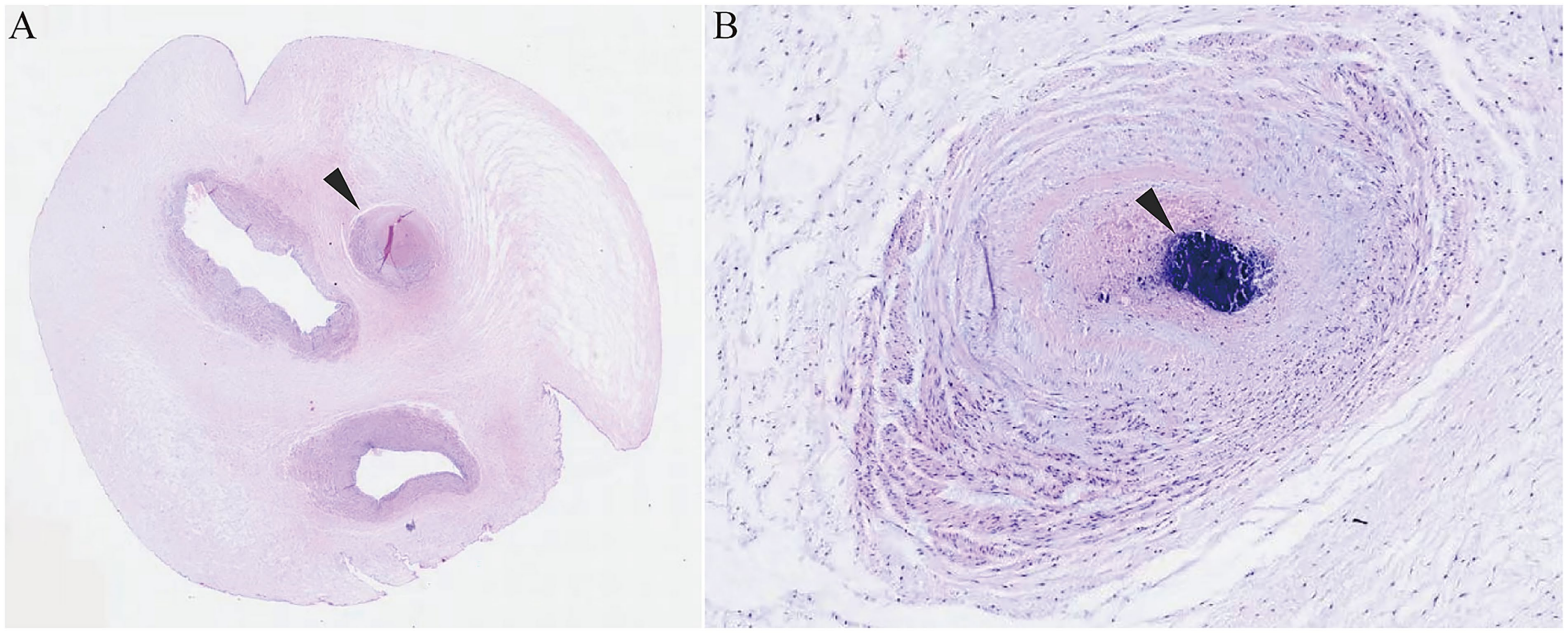
Histopathological examination of the umbilical cord. **(A)** Three vascular lumens with the arrow indicating the occluded umbilical artery lumen. **(B)** Hematoxylin staining showing intraluminal thrombosis with vascular wall collapse. The black arrow indicates the location of the thrombosis.

**Figure 3:**
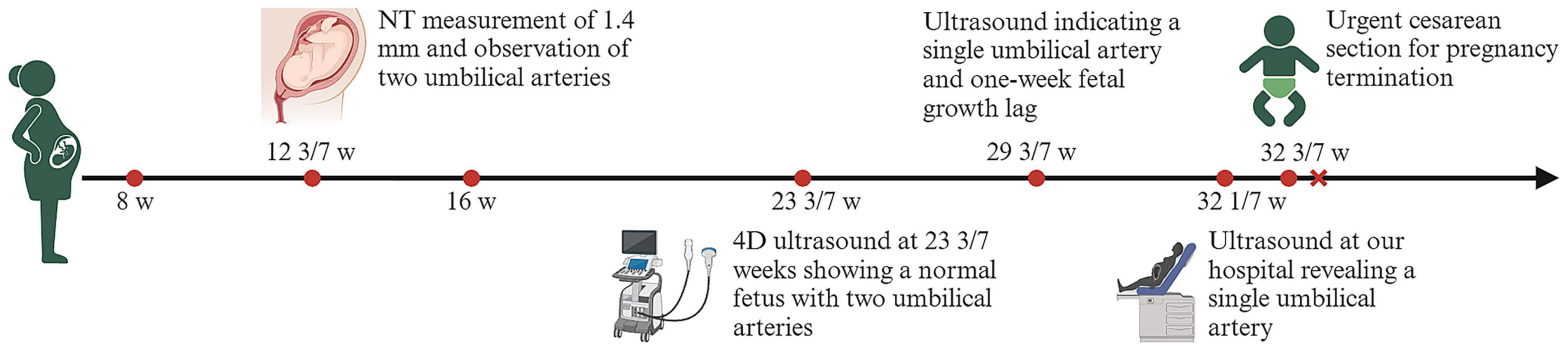
The timeline of ultrasound findings and clinical interventions during the pregnancy. Ultrasonographic measurement of fetal NT is used in prenatal screening for trisomies 21 and 18 and other conditions. NT, Nuchal translucency.

A 32-year-old woman, gravida 2 para 1, with no personal or family history of thrombosis, underwent regular prenatal care during her pregnancy. She was classified as low-risk, with a normal first-trimester screening including nuchal translucency (NT) measurement of 1.4 mm (a sonographic marker for chromosomal abnormalities like trisomy 21) (9) and a mid-trimester four-dimensional ultrasound at 23 3/7 weeks showing a normal fetus with two umbilical arteries (Figure 1A). A 75-g oral glucose tolerance test was within normal limits, and coagulation profiles were unremarkable. At 29 3/7 weeks, an external hospital ultrasound suggested a SUA and fetal growth lagging by 1 week, but no intervention was initiated as fetal movements were normal. At 32 1/7 weeks, she presented to our hospital, where ultrasound revealed a SUA (Figures 1B,C), FGR (below the 10th percentile for gestational age), and abnormal umbilical cord coiling (Figure 1D). Although current ultrasound showed no definitive signs of UAT, the confirmed presence of two umbilical arteries at 23 3/7 weeks (Figure 1A) led to admission with provisional diagnosis of UAT and FGR. Laboratory tests, including coagulation profiles, autoimmune antibodies, and platelet counts, were normal. Treatment included low-molecular-weight heparin, dexamethasone for fetal lung maturation, and magnesium sulfate for neuroprotection. Repeat ultrasound 2 days later showed reduced umbilical artery flow, decreased middle cerebral artery pulsatility index (PI) (1.52 < 10th percentile) (10) (Figure 1E), and hypercoiled cord. An urgent cesarean section was performed. After delivery, a SUA was observed, with hypercoiling of the umbilical cord and localized color changes (Figure 1F). The umbilical cord exhibited over 50 coils and measured about 90 cm in length (umbilical coiling index 0.57 coils/cm, >0.319 diagnostic threshold for hypercoiling) (11) (Figure 1F). The placenta exhibited a circumvallate morphology with a marginally inserted umbilical cord. The male newborn, weighing 1,490 g, achieved Apgar scores of 10/10 at 1 and 5 minutes. He was subsequently transferred to the neonatal intensive care unit (NICU) due to respiratory distress syndrome (RDS), very low birth weight (VLBW), and hypoglycemia. Arterial blood gas analysis showed a pH of 7.27 and PO2 of 53 mmHg. The infant received non-invasive positive pressure ventilation for 3 days, high-flow nasal cannula for 2 days. Moreover, the infant developed hyperbilirubinemia and was treated with phototherapy for jaundice. He was discharged after 27 days with a weight of 2,300 g, showing no complications. Placental pathology confirmed thrombosis in one umbilical artery with muscular necrosis and luminal occlusion (Figure 2A), along with hypercoiling (Figure 2B). Follow-up assessments indicated normal developmental progress in the infant, and no abnormalities were observed in his mother. During postnatal consultations, the mother expressed gratitude, stating: “I’m recovering very well and deeply appreciate the expert care we received throughout this challenging pregnancy.” The timeline of ultrasound findings and clinical interventions during the pregnancy is illustrated in Figure 3.

## Discussion

UAT is a rare obstetric complication, with an incidence of approximately 0.08%. Postmortem fetal examinations reveal a prevalence of 0.1%, rising to 0.4% in high-risk pregnancies (12). The umbilical cord serves as the vital connection between the mother and fetus, and UAT can severely compromise fetal blood and oxygen supply, leading to FGR, fetal distress, or even sudden intrauterine demise (1). Neonatal outcomes are often poor. This case report presents a rare instance of UAT initially misdiagnosed as SUA at a referring hospital. The diagnostic transition from SUA to UAT, accompanied by severe umbilical cord torsion (over 50 complete twists), highlights critical challenges in prenatal ultrasound interpretation. Through multidisciplinary evaluation and timely intervention, the accurate diagnosis was established, leading to appropriate obstetric management and ultimately favorable outcomes for both mother and neonate.

The exact pathogenesis and etiology of UAT remain unclear. Usually thrombosis is associated with three factors: hypercoagulability, endothelial injury, and stasis (13). Pregnancy itself induces a hypercoagulable state, increasing thrombotic risk by 4–5 times compared to non-pregnant states (14). Maternal or fetal hypercoagulability, whether acquired or congenital, is a significant risk factor for UAT (14–16). Studies suggest that the presence of maternal autoimmune antibodies (ANAs) increases thrombotic risk due to their association with inflammation and atherosclerosis, potentially contributing to vascular endothelial damage (14, 17–18). Furthermore, endothelial injury may result from infections or invasive procedures such as intrauterine transfusions or umbilical vein punctures (1, 7). Donepudi and Moise (6) reported a case of umbilical artery thrombosis following five intrauterine transfusions for fetal Rh hemolysis. Additionally, mechanical or anatomical obstructions, such as cord knots, hypercoiling, or velamentous cord insertion, can lead to stasis (19). Redline identified abnormal cord length, excessive torsion, and abnormal cord insertion as common cord abnormalities in UAT (5). Similarly, Li et al. (20) reported that 12 out of 18 UAT cases involved hypercoiling. A literature review of 3,615 cases from 1966 to 2003 found that a cord coiling index > 0.3coils/cm was associated with adverse pregnancy outcomes (21). The exact mechanism of hypercoiling remains unclear, but it likely increases vascular compression, alters hemodynamics, and promotes stasis, thereby elevating thrombotic risk. Moreover, Wu et al. showed maternal gestational diabetes mellitus (GDM) and fetal umbilical cord abnormalities are independent risk factors for UAT (1). In this case, the absence of traditional thrombotic risk factors, such as GDM or infection, suggests that excessive cord length (90 cm) and torsion (over 50 coils) were the primary contributors. However, the temporal relationship between thrombosis and torsion remains uncertain and warrants further investigation.

Prenatal diagnosis of UAT remains challenging, with ultrasound being the primary tool. However, differentiating UAT from SUA is difficult, and approximately 25% of cases are confirmed postnatally through histopathological examination (8, 22). Ultrasound can identify the number of umbilical arteries near the fetal bladder, but the absence of one artery is often mistaken for SUA, especially without prior evidence of a two-vessel cord. SUA is typically identified in early to mid-pregnancy and is associated with structural or chromosomal abnormalities, whereas UAT usually occurs later and focuses on fetal prognosis (8). Li et al. (20) reported that 16 out of 18 UAT cases were misdiagnosed as SUA, leading to six fetal deaths. The “orange peel sign” has been proposed as a characteristic ultrasound finding for UAT (23), while compensatory dilation of the remaining umbilical artery without significant venous changes is indicative of SUA (24). In this case, the external hospital’s failure to investigate the absence of one artery at 29 weeks delayed diagnosis. Fortunately, a thorough review of prior ultrasounds at 32 weeks led to the correct diagnosis and timely intervention, preventing adverse outcomes. Therefore, when a SUA is detected, a detailed review of prior imaging and clinical history is essential. Conversely, an identical presentation in another case resulted in intrauterine fetal demise at 24 weeks’ gestation (8). This finding underscores the diagnostic imperative to differentiate UAT presenting as SUA during early gestation.

UAT can severely disrupt fetal oxygen and blood supply, increasing the risk of hypoxia, FGR, and sudden intrauterine demise. However, there is no consensus on the management of UAT, and treatment must be individualized (2). For near-term pregnancies, cesarean delivery is relatively straightforward, but decisions are more complex for earlier gestations. Studies suggest that for pregnancies <32 weeks, close outpatient monitoring may be appropriate if fetal status is stable, while active intervention and delivery planning are recommended for those >32 weeks (1). Iatrogenic preterm delivery may be unavoidable, but it can prevent the severe adverse pregnancy outcome of sudden intrauterine fetal demise. Oliveira et al. (25) reported a case of UAT with severe FGR managed conservatively until 34 weeks. Furthermore, Zhu et al. (26) advocated for delayed delivery under close monitoring unless abnormalities arise. Conversely, some studies recommend immediate cesarean delivery upon diagnosis of UAT in the third trimester (27, 28), though evidence supporting this approach is limited. The risk prediction model developed by Wu et al. indicates that a gestational age of less than 34.8 weeks and the presence of cord abnormalities are significantly associated with adverse clinical outcomes (1). Low-molecular-weight heparin (LMWH) is commonly used for UAT due to its efficacy and safety in thrombotic disorders (29). Jiang et al. (30) reported that LMWH extended the average gestational age at delivery from 29 to 36 weeks in 10 cases. In this case, the lack of timely diagnosis and anticoagulation at 29 weeks likely contributed to further fetal growth restriction by 32 weeks. Compensatory dilation of the unaffected artery may render umbilical artery systolic/diastolic (S/D) and PI values less reliable for assessing fetal status (31), whereas the middle cerebral artery PI, reflecting the “brain-sparing effect,” is more indicative of fetal hypoxia (32). In the present case, following admission, the patient was treated with LMWH for anticoagulation, magnesium sulfate for fetal neuroprotection, and dexamethasone to promote fetal lung maturation. Fetal monitoring included twice-daily non-stress tests and daily doppler ultrasound assessments of umbilical artery flow and middle cerebral artery PI. Ultrasound findings revealed hypercoiling of the umbilical cord and a reduced middle cerebral artery PI, suggestive of chronic fetal hypoxia. Although fetal movements and cardiotocography showed no signs of acute fetal distress, the decision was made to proceed with early delivery to avoid the risk of sudden intrauterine demise. Following completion of the fetal lung maturation course, a cesarean section was performed, resulting in the delivery of a neonate with vigorous condition.

Notably, our case has certain limitations. First, neonatal coagulation studies were not performed due to concerns about blood volume, leaving the possibility of fetal coagulopathy unexplored. Second, although the neonatal outcome in this case was favorable at discharge, long-term follow-up data were not available. The potential long-term effects of UAT on the infant’s development, particularly neurological outcomes, remain unclear. Future studies should include extended follow-up periods to assess the impact of UAT on childhood development and health.

## Conclusion

UAT can lead to sudden and severe adverse maternal and neonatal outcomes. Early recognition and timely intervention are critical to optimizing outcomes. Individualized management based on gestational age, umbilical cord abnormalities, and fetal middle cerebral artery doppler findings can significantly improve maternal and neonatal prognosis. This case highlights the importance of multidisciplinary care, involving obstetricians, neonatologists, and ultrasound specialists, in the timely diagnosis and management of UAT. Further research is needed to elucidate the underlying mechanisms of UAT and to develop more effective diagnostic and therapeutic strategies.

## Data Availability

The original contributions presented in the study are included in the article/supplementary material, further inquiries can be directed to the corresponding author/s.
